# 

**Published:** 1887-04

**Authors:** 


PAGE.

ORIGINAL COMMUNICATIONS — Organic Diseases of the Œsophagus. Thomas Walley, F.R.C.V.S. ........ 103
Anasarca. Rush Shippen Huidekoper, M. D. ........ 122
Elasticity as a Conservative Force in the Animal Organism. T. Wesley Mills, M. A., M. D. ........ 132
On some Tæniæ of the Quadrumana. William S. Gottheil, M. D. ........ 136
The Veterinary Service of the United States Army. R. W. Shufeldt, M.D., C. M. Z. S., Etc. ........ 138
A few notes on some of the Principal Centers of Veterinary Education in England and on the Continent. A. W. Clement, V. S. ........ 144
NOTES ON THE ANATOMY OF THE INDIAN ELEPHANT.
I. Some Observations on the Brain. Prof. Henry C. Chapman, M. D. ........ 149
II. Anatomy of the Posterior Extremity. Harrison Allen, M. D. ........ 153
III. Notes on the Lungs and Digestive Apparatus. R. S. Huidekoper, M.D. ........ 156
Lanoline. Dr. Alexander Glass ........ 159
Umbilical and Ventral Entero-epiplocele, with a report of three cases successfully treated. Daniel D. Lee, M. D. V. ........ 161
"Barsati," or Atrophic Carcinoma. Richard W. Burke, V. S., A. D. V. ........ 164
Jean Baptiste Auguste Chauveau ........ 174
EDITORIAL DEPARTMENT ........ 176
CASE DEPARTMENT ........ 183
SELECTIONS FROM FOREIGN PERIODICALS ........ 188
PROCEEDINGS VETERINARY MEDICAL SOCIETIES ........ 190
REVIEWS ........ 200

